# Supplementary material for: “Buruli ulcer and leprosy, they are intertwined”: Patient experiences of integrated case management of skin neglected tropical diseases in Liberia
Source: PLoS Negl Trop Dis. 2020 Feb 5;14(2):e0008030. doi: 10.1371/journal.pntd.0008030 (PMC7001903; doi:10.1371/journal.pntd.0008030)
Supplement: Attachment — Submitted filename: comments Plos NTDS.docx [file pntd.0008030.s001.docx]

“Buruli ulcer and leprosy, they are intertwined”: patient experiences of integrated case management of skin neglected tropical diseases in Liberia

COMMENTS

The authors have done a good job by investigating the perception of patients, which has contributed to knowledge by being the first study, but reporting the findings seems to be a bit difficult. A review of the manuscript by someone proficient in English language would have helped.

1-lines 115-116 Geographic coordinates of Nimba County should be included in describing the study location.

2-lines 104-105 “Towards establishing an centre……’’ please rephrase to “Towards establishing a centre……’’

3-line 126 “Patients arrive to the Centre referred by members of the community…..’’ please rephrase.

4- line 127 “laboratory diagnostics are performed…..’’ change to “laboratory diagnoses are performed…..’’

5- lines 128-129 “Most cases are managed as in-patients receiving long term care, and cohabit and receive care as a single group with shared facilities and health workers’’ please restructure the sentence to make it easily understood.

6-line 130 “Those with sufficient mobility living….’’ Please rephrase.

7-line 131 “lodging and treatment for tuberculosis care…’’ change to “lodging and treatment for tuberculosis patients….’’

8- line 143 “We sought 20 patients with a diagnosis of BU or leprosy who had received care……’’ please rephrase using your abstract as a guide; BU (n=10) and Leprosy (n=10)

9-line 144 “Centre for at least two weeks immediately prior to data collection…’’ Change to “Centre for at least two weeks just before the commencement of data collection…..’’

10-line 145 “cognitive impairment, or underage (<18) patients without the presence of a guardian.’’ Change to “cognitive impairment, and underage (<18) patients without the presence of a guardian.’’

11- line 149 “Potential participants were selected by the researcher (MP)…’’ state the meaning of the abbreviation ‘MP’. If it’s a person’s name, please remove it.

12-line 158 “at the Centre….’’ Change to “at the centre…’’

13-line 159 “by the first author (MP)….’’ Should be removed.

14- line 160 “To obtain rich data….’’ Change to “To obtain good data…’’

15-line 166-167 “Observations, perceptions, and experiences interacting with patients and staff were collected as daily field notes jotted down in a paper notebook or recorded as voice memos….’’ Should be changed to “Observations, perceptions, and experiences shared while interacting with patients and staff were collected as daily field notes jotted down in a notebook or recorded as voice memos….’’

16-line 172 “thus ensuring data from a different…’’ change to “thus obtaining data from a different…’’

17- line 177 “lesions of patients were collected during the..’’ change to “lesions of patients were documented during the…’’

18-line 182 “what they saw, thought, and experienced aimed to trigger additional meanings than those….’’ Change to “what they saw, thought, and experienced were used to trigger additional meanings than those….’’

19-line185 “Data analysis occurred simultaneously to data collection..’’ change to “Data analysis occurred simultaneously with data collection…’’

20-line 206 write full meaning of the abbreviation “LSHTM’’

21-line 207-208 “Those who expressed interest to participate underwent written informed consent..’’ change to “Those who expressed interest to participate gave their informed consent….’’

22-line 210 change “patient’s ” to “patients’ ”

23-line 213 “as a reimbursement for their…” change to “as a token for their….”

24-line 220-221 “participant characteristics are described in Table 1.” Change to “participants demographic details are described in Table 1.”

25-lines 222-224 “Here we present results in three sections, covering disease identities and inter-disease stigma, patient experiences of receiving integrated case management interventions, and experiences of rehabilitation in addressing self-stigma, anticipated stigma and disability.” Rephrase to “Our results are in three categories; (1) disease identities and inter-disease stigma, (2) patient experiences of receiving integrated case management interventions, and (3) experiences of rehabilitation in addressing self-stigma, anticipated stigma and disability

26-line 227 “the Centre….” Change to “the centre..” Also make the correction throughout the manuscript.

27-line 230 “made others fear approaching the….” Change to “made others scared of approaching the…” Also “The recent insertion.. ..” change to “The recent inclusion…..”

28-line 231 “facility caused fear among newcomers…” change to “facility caused some apprehension among newcomers….”

29-line 251 “which manifested as experiences of fear of acquiring…” rephrase to “which manifested as a result of fear of acquiring….”

30-line 253 “This sometimes motivated them to use…” change to “This sometimes makes them use…”

31-lines 253-255 “This sometimes motivated them to use outpatient rather than the integrated inpatient care and avoid common bathrooms and mosquito bites, both thought to carry a risk of leprosy transmission:” Rephrase to “This sometimes makes them use outpatient rather than the integrated inpatient care unit. They also avoid the use of common bathrooms and are even scared of mosquito bites in the centre, because of the notion that both could lead to transmission of leprosy’’.

32-lines 261 “with leprosy also expressed initially avoiding..” change to “with leprosy also avoided…”

33-line 268 “in which information about….” Change to “where information about…”

34-line 269 “This knowledge was present among some…” change to “This knowledge evident among some…”

35-line 272-273 “Morning sessions also appeared to favour a sense of community and facilitated relationship-building among people with different diseases.” Rephrase to “Morning sessions also facilitated relationship-building among people with different diseases thereby encouraging communal living.”

36-line 273-274 “Shared demographic features, for example, sometimes helped people bridge biomedical differences…” change to “Having common attributes also helped in bridging the apprehension between patients…”

37-lines 274-275 “This was the case of a man with BU frequently seen spending time with a man affected with leprosy…” change to “This was seen in the case of a man with BU who was frequently spending time with a man infected with leprosy…”

38-line 276 “men were francophone and from Guinea, learning English during their time at the Centre..” Rephrase to “men were from Guinea and were learning English during their time at the centre…”

39-lines 281-284 “Similar experiences in which an intertwined community was built on shared experiences of stigma and disability following structures of gender, language and age were also extensively recorded in the field notes. This helped patients from both disease populations to support each other emotionally and instrumentally throughout integrated care.” Rephrase to “Similar incidences were recorded where people infected with either of the diseases became good friends based on having similar attributes like gender, language, and age. This helped in establishing communal living in the centre.”

40-lines 286-290 “Participants widely understood there were differences in the types of medication and wound care required for each disease. Nonetheless, both groups experienced receiving daily medication as the cornerstone of their rehabilitation: tablets and pills were described as powerful and praised for their ability to remove illness and its attached social burden. Medication inspired hope about a future without disease:” Change to “Most participants understood that there were differences in the types of medication and wound care required for each disease. Both groups received daily medication as the cornerstone of their rehabilitation. They all acknowledged that the tablets and pills were effective in treating their disease, thereby lessening its attached social burden. This inspired them to stay.”

41-lines 293-294 “While their one year course of medication was perceived as lengthy, people with leprosy framed completing it as necessary to restore their social identities:” Change to “Though their one year course of medication was perceived as lengthy, people with leprosy understood that completing it was necessary if they want to restore their social identities.”

42-lines 304-307 “Dressing and wound care of skin ulcers were provided simultaneously to all people affected by BU and a minority of those with leprosy. Both groups perceived wound cleaning and dressing as healing via taking away germs, replacing foul smelling bandages, and avoiding flies….” Change to “Dressing of skin ulcers were done for both patients (mainly BU) at the same time. This was perceived by patients as being very important if healing is to occur because re-dressing removes germs, replaces foul smelling bandages, and also helps in preventing flies from having contact with the ulcers…”

43-line 313 “ulcers received manual debriding consisting of removal …” rephrase to “ulcers underwent manual debriding involving removal…”

44-line 314 “traditional medicine approaches…” rephrase to “traditional medication..”

45-line 323 “accumulations of fluids, and pain management was limited to injection of intramuscular…” rephrase to “accumulations of fluids, while the pain was managed by intramuscular injection of …”

46-line 325 “observed during fieldwork and present in participants’…” rephrase to “observed during fieldwork and were documented in participants’…”

47-line 326 “experiences shaped attitudes of occasional refusal or resistance to participate in wound care…” rephrase to “experiences contributed to occasional refusal or reluctance in participating in wound care …”

48-line 338 “often arrived to the Centre..” rephrase to “often arrive the centre…”

49-line 345 “which could be coordinated via referral..” rephrase to “such patients were referred to..”

50-line 346 “staff mentioned referrals were hindered by resistance..” rephrase to “staff mentioned that referrals were affected by resistance….”

51-line 347 “the Centre due to fear of transmission..” rephrase to “the centre due to fear of disease transmission…”

52-line 354 “BU was a recurring..” rephrase to “BU was common….”

53-line 357 “favourable experiences..” change to “good reports..”

54-line 358 “participant’s” change to “participants’

55-line 369 “emphasised” change to “reported”

56-line 370 “productivity” change to “activities”

57-lines 370-372 “as well as fear that their bodies would be permanently disabled, was an important motivator for patients to master these mobility devices or reduce their dependence on them…” Restructure the sentences to bring out what you mean clearly.

58-lines 375-377 “Weekly medical rounds which focused on individual assessments emphasizing completion of chemotherapy and continuous wound care tended to neglect physical rehabilitation plans to prevent lasting functional disability..” Change to “Weekly medical rounds which focused on individual adherence to chemotherapy and continuous wound care, neglected physical rehabilitation plans to prevent lasting functional disability…”

59-line 378 “important” change to “severe”

60-line 379 “incompatible with productivity:” change to “resulting in low productivity..”

61-line 383 “enabled” change to “provided”, “progressiveness of these” should be removed.

62-line 393 “this” should be removed.

63-lines 393-396 “had the effect of blurring the lines between disease identities, not only shaping unique configurations of inter-disease stigma, but also linking patient groups within a single patient community”... Rephrase with simple sentences to pass your message across.

64-lines 397-398 “in the collective imaginary” .This statement is not clear. I suggest you remove it.

65-line 408 reference [22]. Is it in agreement with the statement “stigma and disability for both diseases, slowly rehabilitating people’s “spoiled” identities..” ? Restructure to reflect the reference.

66-line 408 “experiences” change to “outcomes”

67-lines 411-413 “These experiences are expressions of actionable implementation challenges that should be considered in the design of patient-centred approaches”. Rephrase to make the message clear.

68-line 423 “experiences of receiving” change to “outcomes of”

69-line 424 “unveiled” changed to “revealed”

70-line 426 “experiences facilitated” change to “needs” and “harmonizing experiences of integration” change to “helped in harmonizing the integration”

71- line 430 “facilitate” change to “encourage” and “thus enhancing their feasibility” should be deleted.

72-line 431 “study” change to “findings” and “might have an unanticipated effect on” change to “affected”

73-line 434 “conceive” change to “perceive”

74-line 440 “alternate condition” change to “two conditions”

75-lines 440-442 “reinforce that the integration of case management for skin NTDs should anticipate this phenomenon in mixed patient communities” change to “is similar to the Nepal study, regarding stigma within the two groups of patients, and this phenomenon should be expected in other mixed patient communities.”

76-line 445 “and delivered” change to “and should be delivered”

77-line 446 “of” change to “from”

78-line 449 “opportunity” change to “need”

79-line 453 “negative experiences” change to “some challenges” and “as a consequence of a” change to “in a”

80-line 454 “including” change to “which include”

81-line 455 “Liberia’s history” change to “Liberia’s history of war”

82-line 456 “context shape” change to “status resulted in”

83-line 458 “these particular implementation” change to “other smaller”

84-line 460 “including secondary…” change to “which include

85-line 462 “recurrence” change to “adverse consequences” and “Additionally, holistic approaches to” change to “Again”

86-line 463 “that focus” change to “that also focus”

87-line 465 “This includes” change to “This should include”

88-line 469 “do not” change to “did not”

89-line 470 “exploring” change to “investigating”

90-line 473 “Second” change to “Secondly”

91- line 475 “elicited” change to “shared”

92-lines 477-478 “study further supports the role of applied social sciences in locating people’s experiences at the forefront of programme design and implementation” Rephrase to “study further supports the need for applied social sciences methodologies to be included at both the planning and implementation stages of such programs designed to obtain information about experiences of people infected with such diseases”.

93-line 480 “context” change to “study”

94-line 483 “configurations: future social…” change to “configurations. Future social….” And “context” changed to “area”

95-line 485 “including” change to “like”

96-line 486 “complex cases of BU.” Change to “complex cases of BU, and their impact.” And “Further” should change to “More”

97-line 493 “ongoing support to conduct…” change to “support in conducting…”

98-line 496 “helped to anticipate potential risks.” Rephrase to “helped us in knowing what to expect.”
