# Supplementary material for: “Buruli ulcer and leprosy, they are intertwined”: Patient experiences of integrated case management of skin neglected tropical diseases in Liberia
Source: PLoS Negl Trop Dis. 2020 Feb 5;14(2):e0008030. doi: 10.1371/journal.pntd.0008030 (PMC7001903; doi:10.1371/journal.pntd.0008030)
Supplement: Attachment — Submitted filename: Response to Reviewers PLOS NTDs.docx [file pntd.0008030.s002.docx]

PLOS Neglected Tropical Diseases

15 November 2019

Dear Editors,

We are pleased to resubmit an improved version of our manuscript **“*Buruli ulcer and leprosy, they are intertwined*: patient experiences of integrated case management of skin neglected tropical diseases in Liberia (PNTD-D-19-01195)”** for publication in PLOS NTDs.

Please find below our replies to the comments kindly provided by peer reviewers:

1. **“Reviewer #1: The research is largely focused on the experiences of the patients, and the authors have described this as a field-based qualitative case series. However, it is not clear the rationale for selecting this qualitative design approach. As there are several other qualitative approaches that could have been adopted, the authors may wish to state their reason(s) for this choice.”**

We appreciate the opportunity to justify our chosen methodology. Our aim was to document and explore patient experiences of integrated case management to inform future programmatic improvement. Our qualitative study included in-depth interviews with patients (“cases”) with BU or leprosy in a referral facility during a defined period of time. In this context, patients received a complex intervention package. We chose to use the “case series” descriptor for our study design since this adequately represented the approach of briefly following a group of patients receiving care in a single facility. This also facilitated within-case and across-case analysis, and provided the opportunity of understanding programmatic patient outcomes, as exemplified with Case 014 who died due to BU-related complications during the fieldwork. We have now included the following justification in the opening paragraph of the Methods section (lines 123-24): *“This methodological approach was chosen on the basis of conducting patient-centred research in a clinical context.”*

Given the cultural and social aspects of NTDs, we decided to ground the identification of themes in the experiences of patients. We have now included the following justification in the data analysis section of our manuscript (lines 217-22): *“We reported our findings as patient experiences to avoid biomedical interpretations that might further remove future programming from users’ needs. While other approaches, such as emphasising experiences of health workers and operational challenges for programming are also needed, trustworthiness in the exploration of socio-cultural themes such as stigma, disability, and rehabilitation required a patient-centred approach.”*

2. **“Reviewer #1: Although the authors have mentioned the overall aim of the study, ‘The aim of this qualitative study was to explore the experiences of in-patients affected by BU and leprosy in Liberia receiving integrated case management at Ganta rehabilitation centre.’, the quality of the study will be further strengthened if the specific research question(s) are stated. The research question(s) would help readers to know the specific issue(s) the authors will be addressing in their research.”**

We appreciate this suggestion. Our *a priori* research question was broad and stated in the same way as our overall study aim: *“What are the experiences of integrated case management among patients affected by leprosy and Buruli ulcer in Ganta, Liberia?”*. However, given the primarily inductive/exploratory nature of our inquiry, some of our most salient themes such as inter-disease stigma and disease identities emerged during the analysis stage. This methodology has been clearly detailed in the data analysis section (line 204-07): “*We used a first cycle of coding to develop a tentative codebook and identify emerging themes, using primarily an inductive approach*”, and in line 213 onwards: *“The matrix was rearranged and recompleted iteratively throughout the analysis process, as new themes emerged and others converged into larger themes”.* We agree that readers would benefit from a clear statement of the specific issues and themes addressed in our research, which are now clearly stated at the beginning of the results section (lines 246-50): “*Our results are presented in three sections: (1) disease identities and inter-disease stigma, (2) patient experiences of receiving integrated case management interventions, and (3) experiences of rehabilitation addressing stigma and disability”*

3. **“Reviewer #1: In addition, there is need for the authors to provide a clearer description of how integration was implemented in this setting, as this would be helpful for replicability of the intervention in a similar setting.”**

We appreciate this observation. We have now included two additional sentences in the “Study setting” sub-section of our manuscript indicating how integration was implemented (lines 132-36): *“Based on WHO-driven initiatives to integrate case management for skin NTDs, the Ministry of Health decided to upgrade the centre’s capacity, incorporating BU care to leprosy care since 2015. Following the National Strategic Plan for the Case Management of NTDs, centre staff was trained in laboratory diagnostics and care for BU”.* This includes a reference to Liberia’s national plan for case management of NTDs, which can be accessed online and further details the national policies, strategic framework and implementation plan that led to integration of care.

4. **“Reviewer #1: Although the authors did give an indication of their inclusion and exclusion criteria, they however did not state the reasons why these where adopted. The reasons for adopting these criteria need to be clearly stated.”**

We appreciate this observation and have clarified accordingly (lines 159-63): *“We included patients who had received care at the centre for at least two weeks to ensure they had experienced case management activities. We excluded patients with cognitive impairment since this might make interviewing difficult, and underage (<18) patients without the presence of a guardian since they could not legally provide written informed consent.”*

5. **“Reviewer #3: The methods section needs to be organized properly. The design of the study was not stated but rather a description of what was done. Similarly, the authors did not clearly document how the participants were selected. Describe the selection procedure”**

We appreciate this comment. We conducted a qualitative case series. Our study design was clearly stated, and has now been justified as suggested by reviewer #1 (line 121-25): *“We conducted a field-based qualitative case series of patients with BU and leprosy receiving care at the Ganta Tuberculosis & Leprosy Rehabilitation Centre in Nimba County, Liberia. This methodological approach was chosen on the basis of conducting patient-centred research in a clinical context”*

We have now described the participant selection procedure in more detail (lines 164-70): *“During the first week of field work, potential participants were selected by a researcher (MP) during informal conversations or via clinical chart review to identify patients that met the inclusion criteria. We invited patients to participate in the study during delivery of chemotherapy and wound care services, or other activities in common areas in the centre. We purposively sampled participants across the range of ages, genders, languages and counties in the sample to ensure maximum variability of experiences. Health workers did not have a role in selecting potential study participants.”*

6. **“Reviewer #1: The data analysis process is described in some detail, the authors mention they applied both inductive and deductive approaches to data analysis. From the article, it is not very clear why both approaches were adopted, what they mean how they were applied.”**

We appreciate this comment and have clarified accordingly. It is frequent in qualitative analysis to approach coding using a mixture of inductive (data-driven) and deductive (based on pre-existing constructs) strategies (Miles & Huberman, 2014). While we used both, our thematic analysis was primarily inductive. We have clarified in lines 205 onwards: *“We used a first cycle of coding to develop a tentative codebook and identify emerging themes, using primarily an inductive approach. Deductive coding based on a literature review also contributed to initial codebook development.”*

7. **“Reviewer #3: Some areas of the results were methods. Line 221 to 225 should be part of the methods section”**

We appreciate this comment. In our Methods section we have described data collection procedures with detail. The opening paragraph of our Results section indicates the results of our data collection, including the number of interviews to patients, health workers, and field notes used for our analysis. We consider it is best practice to report the results of data collection in the Results section.

8. **“Reviewer #1: In the limitations section, the authors should consider mentioning the limitations of using a word processing software for transcription. Also, the authors may need to include comments on how rigour was ensured, such as trustworthiness, truth-value, applicability, consistency, and neutrality.”**

We appreciate this comment. We believe Reviewer #1 might have understood our use of a word processing software for transcription as using an automated transcription service. Transcription was done by the research team listening to the audio recordings and typing sections in English verbatim in Microsoft Word. We have now rephrased this more clearly in line 185: *“The lead researcher’s observations, perceptions, and experiences of interacting with patients and staff were collected as daily field notes or recorded as voice memos, and transcribed at the end of each day.”*

9. **“Reviewer #1: The authors triangulated findings with health workers and their observations, did the patients themselves review and comment on the analysis as well?”**

We appreciate the reviewer’s interest on this matter. Patients did not review or comment on the final analysis, since this was completed after fieldwork ended. However, since a large portion of data analysis occurred simultaneously to data collection, in-depth probing about emerging themes was incorporated in our flexible semi-structured interview guidelines, particularly on themes of inter-disease stigma and physical rehabilitation. In this way, new participants commented and provided their own experiences in juxtaposition with those of prior participants. This has now been detailed in the “Interviews” subheading of the methods (line 175): *“We continued to refine interview guidelines during data collection to include additional probing on emerging themes.”*

10. **“Reviewer #3: Conclusion was not very clear on the objectives but rather mixed with future research potentials. It’s important that the authors conclude on the objectives of the study before making recommendations based on their findings”**

We appreciate this observation and have reworded our conclusion to better match our study objective. The first paragraph of the conclusion relates to the study objective of exploring patient experiences of integration (lines 505-10): *“Based on patient experiences, integrating case management of leprosy and BU in this context appears to be effective in addressing the needs of people affected by these diseases, particularly stigma and disability. Integration was experienced positively by patients, but reshaped discourses on disease identity around unique inter-disease stigma configurations. Patients experienced consequences of limited resources, such as lack of support to reduce lasting disability, and poor management of BU complications.”*. The final paragraph refers to recommendations and future directions for research.

11. **“Reviewer #1: Line 365 – 367 “A decent man, he’s working! The whole fingers 365 correct, the toes correct, everything on him is fine! No problem, you can’t even know he had leprosy."(Case 006, MBL, M, 21). It is good to see that the authors mentioned the positive impact of early treatment.  In the discussion section, the authors might want to consider further emphasising that early diagnosis and treatment are crucial to minimizing morbidity, costs and prevent long-term disability in both leprosy and buruli ulcer patients. This could be a key driver for country programmes to conduct integrated active case finding interventions.”**

We appreciate this suggestion and agree that early case finding is fundamental to minimise skin NTD morbidity. We have additional data supporting experiences of integrated case detection and referral that we are preparing to submit as a separate manuscript for publication, including explorations on the use of traditional medicine and the quest to curative biomedical treatment. However, the focus of our current submission was not on active case finding at the community level, but on case management at the facility level, and we prefer to circumscribe our discussion to this topic.

12. **“Reviewer #1: Line 133 ‘People with leprosy receive one year of multidrug therapy (rifampicin, clofazimine and dapsone)’. this statement is unclear as leprosy patients were not classified, and not all leprosy patients receive treatment for 12 months. Multibacillary (MB) leprosy patients are treated for 12 months, while paucibacillary (PB) leprosy patients are treated for 6 months.”**

Thank you for this comment. We have reworded this sentence to improve accuracy, lines 145-47: *“At the centre, patients with multi-bacillary leprosy, defined as having at least 5 skin lesions, receive one year of multidrug therapy (MDT) with rifampicin, clofazimine and dapsone. People with pauci-bacillary leprosy (<5 lesions) receive MDT for six months.”*

13. **“Reviewer #1: The authors may wish to consider including a table that highlights themes, subthemes etc. as this would provide a succinct summary of the findings and how the themes interact with each other.”**

We appreciate this suggestion. While a table providing a theme overview with a representative quote per theme could be useful, it would imply a significant rearrangement of our results section as it currently stands, which is heavily reliant on the embedded quotes. Instead, we have provided a succinct summary of our findings in the abstract, the first paragraph of the discussion, and in the conclusion of our manuscript. In the interest of space, we believe it is best to keep our current outline, maintaining an in-depth description of our themes and sub-themes in each heading of the results.

14. **“Reviewer #3: The methods section needs to be organized and copyediting done before publishing. An interesting work”**

We appreciate this comment. We have kept the structure and organisation of our Methods section (Study setting and population, In-depth interviews, Field notes, Photography, Data management and analysis, and Ethics). Based on the feedback receive by peer reviewers, we have re-written and copyedited several sub-sections in our Methods section to improve readability.

15. **“Reviewer #3: Overall, an interesting work but some areas need to be worked on and copyediting is required to make the language ease to read. Kindly ensure that abstract brings out the key issues but in the current state it is not the best. Especially the introduction and the conclusion.”**

We appreciate this comment, and have now updated our abstract to better reflect the key issues of our research.

Our updated manuscript also includes other minor modifications, including a revision of our scientific writing, grammar and language, as indicated by Reviewer #2. Many of the suggestions sent as an attached word document have been implemented in the body of the manuscript. We hope you consider our updated manuscript suitable for publication with your journal.

Thank you for your consideration, and we look forward to hearing from you.

Sincerely,

*Mateo Prochazka, MD MSc on behalf of all authors.*

*London School of Hygiene and Tropical Medicine*
